# Robot-Assisted Simple Prostatectomy: Illustration of a Simplified Extraperitoneal Transcapsular Technique

**DOI:** 10.1089/vid.2019.0032

**Published:** 2019-04-08

**Authors:** Pratik M. Gurung, Prabhakar Mithal, Diane D. Lu, Ahmed E. Ghazi, Jean V. Joseph

**Affiliations:** Department of Urology, University of Rochester Medical Center (URMC), Rochester, New York.

**Keywords:** robotic simple prostatectomy, extraperitoneal simple prostatectomy, benign prostatic hyperplasia

## Abstract

***Introduction and Objectives:***Robot-assisted simple prostatectomy (RASP) performed with the extraperitoneal (EP) technique (RASP-EP) minimizes the risk of bowel injury, particularly when bowel adhesions may be expected to be prominent, by negating the need to be in the transperitoneal space. However, there is a perception of its technical difficulty owing to the limited space that can be expanded within the space of Retzius. We aimed to describe, in the accompanying video, the step-by-step approach for a technically proficient procedure.

***Methods:*** From January 2010 to July 2018, 33 consecutive patients who had undergone RASP-EP were identified from our institutional database. Procedures were performed as described stepwise in the accompanying video. In RASP-EP, a 3 cm paraumbilical incision is made, anterior rectus sheath incised, muscle pushed laterally, and the EP space is entered. The EP space is expanded in the retropubic area using a balloon dilator and a blunt ended trocar, enabling the placement of further three ports for robot docking. A transverse capsulotomy, 2 cm from the bladder neck, is performed *a la* Millin's. Prostate adenoma is resected circumferentially. Electrocautery hemostasis is performed. Posterior bladder neck and urethra are sutured onto the prostatic fossa with 2-0 Vicryl. A 22F three-way catheter is placed. Anterior capsulotomy is closed in two layers with 2-0 and 0-0 Vicryl sutures. A drain is left in the retropubic space. Patient is discharged within 1–2 days with the catheter *in situ*, which is then removed 10 days later.

***Results:*** Of the 33 patients, median values were age (68), American Society of Anesthesiology (3), Charlson Comorbidity Index (3), and body mass index (28.5 kg/m^2^). Eight (24.2%) patients had prior abdominal surgeries. Twenty-five (75.8%) patients were catheter dependent. Adjunctive procedures were cystolithotomy (5), umbilical hernia repair (2), and ureteroscopy (1). Median values were operative time (178 minutes), estimated blood loss (200 mL), hemoglobin change (2.8 g/dL), and hematocrit change (9%); only one patient (3.0%) required 1 U transfusion. Median length of stay was 2 days. Clavien–Dindo complications were 0 (21), I (7), II (3), IIIa (1), IIIb (1), IV, and V (0). Median resected prostate weight was 122 g. Incidental prostate cancer was found in three patients (9%); one patient required adjuvant radiotherapy. No patients were catheter-dependent postoperatively; mean postvoid residual was 29 mL (range 0–250 mL). Median follow-up was 4 months.

***Conclusions:*** RASP-EP is a safe and efficacious technique that should form the repertoire of a urologist's armamentarium when dealing with large adenomas, particularly when entry into the peritoneal cavity is to be avoided.

No competing financial interests exist.

Runtime of video: 7 mins 5 secs

## Graphical abstract

**Figure:**
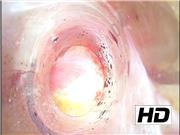

**Figure:**
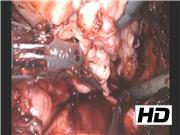

**Figure:**
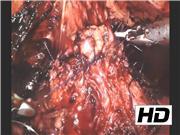

## Video

## Files

**MP4:** 

**PDF:** 

